# Designing and simulating realistic spatial frequency domain imaging systems using open-source 3D rendering software

**DOI:** 10.1364/BOE.484286

**Published:** 2023-05-04

**Authors:** Jane Crowley, George S. D. Gordon

**Affiliations:** Optics & Photonics Group, Department of Electrical and Electronic Engineering, University of Nottingham, Nottingham, United Kingdom

## Abstract

Spatial frequency domain imaging (SFDI) is a low-cost imaging technique that maps absorption and reduced scattering coefficients, offering improved contrast for important tissue structures such as tumours. Practical SFDI systems must cope with various imaging geometries including imaging planar samples *ex vivo*, imaging inside tubular lumen *in vivo* e.g. for endoscopy, and measuring tumours or polyps of varying morphology. There is a need for a design and simulation tool to accelerate design of new SFDI systems and simulate realistic performance under these scenarios. We present such a system implemented using open-source 3D design and ray-tracing software *Blender* that simulates media with realistic absorption and scattering in a wide range of geometries. By using *Blender’s* Cycles ray-tracing engine, our system simulates effects such as varying lighting, refractive index changes, non-normal incidence, specular reflections and shadows, enabling realistic evaluation of new designs. We first demonstrate quantitative agreement between Monte-Carlo simulated absorption and reduced scattering coefficients with those simulated from our Blender system, achieving 16% discrepancy in absorption coefficient and 18% in reduced scattering coefficient. However, we then show that using an empirically derived look-up table the errors reduce to 1% and 0.7% respectively. Next, we simulate SFDI mapping of absorption, scattering and shape for simulated tumour spheroids, demonstrating enhanced contrast. Finally we demonstrate SFDI mapping inside a tubular lumen, which highlighted a important design insight: custom look-up tables must be generated for different longitudinal sections of the lumen. With this approach we achieved 2% absorption error and 2% scattering error. We anticipate our simulation system will aid in the design of novel SFDI systems for key biomedical applications.

## 1. Introduction

**Fig. 1.**
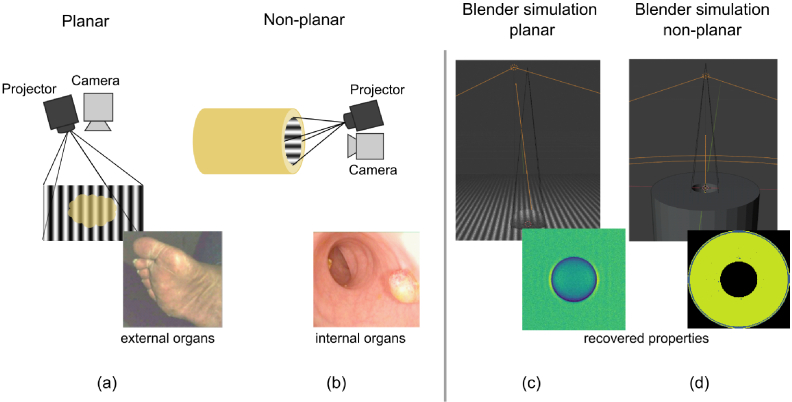
Future SFDI systems, especially those for *in vivo* clinical use, may require significantly different geometries from conventional SFDI: a) conventional ‘planar’ SFDI imaging geometry with projector at a small angle to planar sample, with real-world application of measuring diabetic foot shown in inset [18], b) SFDI operating in a tubular (lumen) geometry, that may be required for use in future endoscopy systems where projection is no longer approximately planar, with example usage for imaging polyps in the colon shown inset [19], c) screenshot of our *Blender* SFDI model applied to a planar geometry, with reconstructed scattering properties of tumour like sample shown inset, d) a screenshot of our *Blender* model applied to a non-planar tubular geometry, with reconstructed scattering properties shown inset.

Optical properties, specifically absorption and scattering, and shape are important potential indicators of cancer within the gastrointestinal (GI) tract [1,2]. Conventional white light endoscopes and capsule endoscopes are the standard method of imaging the GI tract but provide limited information about tissue properties that are hallmarks of a range of potential tumours [3], leading to low five-year survival rates of oesophageal cancer (15% [4]) and colon cancer (63% [5]). SFDI is a well-studied, low-cost imaging technique [6,7], with applications for imaging blood oxygenation [8], burn depth [9], dental caries [10], bowel ischaemia [11], and indicators of cancer [12]. A range of commercial [13] and research [14–16] SFDI systems are now available. However, these existing systems are almost exclusively designed for *planar* imaging geometries, where the sample is uniform in morphology and the camera and projector are located above it at near-normal incidence (Fig. 1(a)). However, many important clinical applications exhibit *non-planar* geometries: for example imaging inside tubular lumen such as the GI tract, blood vessels, biliary system (Fig. 1(b)). SFDI imaging *in vivo* in such organs is challenging due to miniaturisation needs, and because the surfaces are cylindrical, creating non-planar illumination conditions and sample geometries. This means that illumination and imaging may no longer be normal (or nearly normal) to the surface being imaged so different scattering behaviour will be observed [17], and specular reflections will be altered. To aid in the design of novel SFDI systems under these constraints, we have created an SFDI design and simulation tool in the open source 3D modelling software *Blender* (v 2.93) using the built-in ray-tracing engine Cycles (Fig. 1(c) and 1(d)). Cycles is a physically based path tracer, in which randomly generated rays of light are traced from each camera pixel into the scene and can be absorbed, reflected, refracted or scattered, analogous to a Monte Carlo simulation [20]. Cycles simulates volume scattering inside objects using a Henyey-Greenstein Phase function, which is commonly also used in Monte-Carlo simulations of tissue [21,22]. *Blender* has previously been used for three-dimensional shape measurement of additive manufacturing parts with complex geometries [23], for the development of anatomically accurate meshes to use in Monte Carlo light simulations [24], and for the generation of SFDI image data sets to train neural networks [25,26]. By using Blender for both geometry specification (i.e. design) and simulation (via ray-tracing with Cycles), we are able to simulate realistic optical properties and geometries while naturally accounting for realistic features of SFDI systems such as stray light, specular reflections and shadows.

Conventional imaging in the spatial frequency domain consists of projecting a known set of structured illumination patterns onto a sample at a small angle (typically $10^{{}^{\circ}}-20^{{}^{\circ}}$ ) to the normal to minimise specular reflections recorded by the camera [8]. The structured illumination set typically consists of 2D sinusoids at 3 equispaced phase offsets [27] but these results can also be obtained from a single image, termed *single snapshot of optical properties (SSOP)*, by the use of Fourier-domain filtering [28] or convolutional neural networks [29] to separate the AC and DC components. Recent work has also shown the successful use of randomised speckle patterns as an alternative illumination scheme [30].

Typically, the AC and DC modulation amplitudes need to be calibrated with the modulation transfer function (MTF) of the system in order to produce *diffuse reflectance* values that represent the next intermediate step toward obtaining absorption and scattering. Conventional calibration for the MTF is achieved by imaging a reference material of known optical properties and computing diffuse reflectance values for these properties using a light propagation model: Monte Carlo simulation or the Diffusion Approximation [27,31]. The difference between the computed and measured diffuse reflectance is used to infer the MTF, which can then be applied to obtain diffuse reflectance values from the modulation amplitudes of the sample of interest. Finally, the absorption, $\mu_{a}$ , and reduced scattering, $\mu_{s}^{\prime }$ , coefficients are determined via a look-up table (LUT) generated using the chosen light-propagation model. Alternatively, an *empirically derived* LUT relating AC and DC modulation to optical properties can be directly created by measuring a library of materials in comparison to a reflectance standard [32].

SFDI systems can also extract height information via fringe projection profilometry [33,34]. The distortion of the fringe pattern by the presence of the sample can be used to determine sample height and combined with 2D shape gives an estimate of the 3D morphology [12]. This information can be useful in clinical settings for quantifying the morphology and volume of polyps, which is linked to their pathology [2] and proof-of-principle structured illumination systems have been trialled [35].

Developing an SFDI system suitable for determining optical properties and shape in clinical environments, both *ex vivo* and *in vivo*, has many challenges associated with it, such as examining the effect of illumination source placement and determination of optimum illumination patterns. Here, we present a design and simulation system using free, open-source 3D modelling and rendering package *Blender*, that can simulate SFDI for recovery of absorption, scattering and shape. We first show how to use *Blender* to model a customisable absorbing and scattering material using *Blender* material nodes. We then show how to construct a virtual characterisation system for the absorption density, $A_{\rho }$ , and scattering density, $S_{\rho }$ , of this material using two approaches: a double integrating sphere (DIS) [36] and an SFDI system. For both approaches, we validate the accuracy of retrieved optical properties and show how this can be improved by generating an empirically derived LUT from the DIS *in-situ* data. Next, we present two illustrative example cases for our system. First, we show that the simulated SFDI system enables reconstruction of scattering, absorption and shape of planar geometry samples mimicking cancerous and pre-cancerous conditions such as squamous cell carcinoma and Barrett’s Oesophagus respectively. Second, we demonstrate, for the first time, a novel illumination scheme tailored for non-planar, tubular geometries (such as inside a lumen) where the spatial frequency is constant throughout the length of the tube such that the optical properties can be accurately obtained. To improve accuracy, we longitudinally section the tube and create separate look-up tables for each section, a straight-forward task in our system. We show that this customised illumination can detect changes in absorption and scattering properties within a tube of biologically relevant material, providing a potential design for future SFDI systems.

## 2. Methods

### 2.1 Material simulation

Previous work has used a weighted mixture between transparent, absorbing, and sub-surface scattering materials to create a composite material with the desired optical properties [25]. Though this approach works in many realistic operating regimes, it is limited because the sub-surface approximation applies only at surfaces and not in the entire material volume. Here, we therefore model the material using a volume shader, exploiting Blender’s built-in volume absorption and volume scattering functionalities. The absorption and scattering were varied by changing the density parameters of the nodes, $A_{\rho }$ and $S_{\rho }$ respectively. The anisotropy, *g*, in the volume scatter node was set to 0.8. This value is representative of typical anisotropy values measured for tissue at the GI junction [1].

Blender supports tri-colour operation so it can provide physically realistic scattering at green and blue wavelengths if desired. However, we configure the volume scatter, absorption and surface reflectance to be equal in these three bands, simulating a white material. In Blender, this is achieved by setting the colour parameter of the shader nodes to white (RGB = (1.0, 1.0, 1.0)). Further, when capturing image data, we extract the red channel of the RGB colour images. The refractive index of the material is set to n=1.4 by connecting a glass bi-directional scattering distribution function (BSDF) shader node to the surface input of the material. This shader was set to have a surface roughness of 0.5 .

In order to use a LUT generated from a Monte-Carlo simulation or the Diffusion Approximation, the semi-infinite thickness requirement must be met [37]. To set an appropriate thickness for the material to meet this property, a red sphere was placed behind the material with variable $A_{\rho }$ and $S_{\rho }$ properties, and the parameters were varied until the difference in intensity between a 20×20 pixel region within the red sphere boundary and a 20×20 pixel region outside the red sphere boundary was ≤1% . For a material of 2 m thickness, this threshold was achieved for $A_{\rho }>5$ when $S_{\rho }=0$ , and for $S_{\rho }>4$ when $A_{\rho }=0$ . These are therefore the lower bounds of the material parameters in our simulation, but this limitation could be circumvented by using an empirically derived LUT calibrated to a particular physical thickness. The scene was illuminated by a sun light source of strength 10.

Our aim is to create a simulation of an SFDI system with biologically relevant samples, and so we have identified two disease states relevant for detection of cancer in the upper GI tract: squamous cell carcinoma (SCC) and Barrett’s Oesophagus (BO) [38]. For SCC we modelled tumour spheroids using sphere meshes scaled to be 80 mm in diameter (40 mm height from the base material). We note that the *‘scale’* parameter of the object in Blender should be reset when the desired size is reached to ensure proper behaviour with regard to scattering length scales. At 635 nm, the absorption coefficient of SCC is 0.12 mm^−1^, which is much greater than that of healthy oesophageal tissue, 0.058 mm^−1^, and the reduced scattering coefficient of SCC, 0.64 mm^−1^, is less than that of healthy oesophageal tissue, which is typically 0.75 mm^−1^ [39].

To simulate BO, two materials were placed adjacent to one another: one with the optical properties of healthy oesophageal tissue and the other with the optical properties of BO. At 635 nm, the absorption coefficient of BO with mild chronic inflammation is 0.057 mm^−1^, which is similar to that of healthy oesophageal tissue, while the reduced scattering coefficient of BO with mild chronic inflammation, 0.51 mm^−1^ is much less than that of healthy oesophageal tissue [39].

To simulate realistic gastrointestinal imaging, we consider two imaging geometries. The first simulates an ‘up-close’ view of a tumour on the wall of a large lumen and can be approximated by a planar geometry. However, to identify such structures during a typical endoscopy or to examine such structures in a smaller lumen, it is also necessary to consider a tubular geometry with a forward-facing wide field-of-view. We therefore also consider the scenario of an SFDI system pointing down a tube, shown in Fig. 1(b).

To achieve the most physically realistic ray-traced renders in *Blender*, some optimisation of the render settings is required. Within the ray-tracing engine Cycles, the maximum number of bounces a light ray can travel before the simulation terminates can be set. We set this value to 1024, the highest allowed. We found that, for a semi-infinite material simulating healthy oesophageal tissue, halving the maximum number of bounces from 1024 to 512 resulted in a minimal decrease in the AC and DC modulation amplitudes of 0.03% and 0.2% respectively, showing that a limit of 1024 is likely to be sufficient for most important practical cases. The number of samples to render per pixel in the image was set to 1000. Clamping of direct and indirect light, which limits the maximum intensity a pixel can have, was disabled by setting both to 0. Colour management, which is typically used to make visually appealing images but introduces unwanted artefacts such as gamma correction, was disabled by setting the display device to *‘None’*. View transform was set to *‘Standard’* to ensure no extra processing is applied to the resulting images. The sequencer, which sets the the colour space, was set to *‘Raw’* to avoid unwanted colour balancing or further gamma correction. For all images rendered, the camera exposure is adjusted in accordance with *Blender* documentation [40] to avoid saturation while maximising power of detected signal, but the images must then have their intensities corrected by following the equation:

(1) $$I_{\text{output}}(x,y)=I_{\text{render}}(x,y)\times 2^{t_{\text{exposure}}}$$

where $I_{\text{output}}$ is the exposure-corrected intensity we require, $I_{\text{render}}$ is the raw value obtained following the render, and $t_{\text{exposure}}$ is the exposure setting.

### 2.2 Calibration of material optical properties

#### 2.2.1 Double integrating sphere

**Fig. 2.**
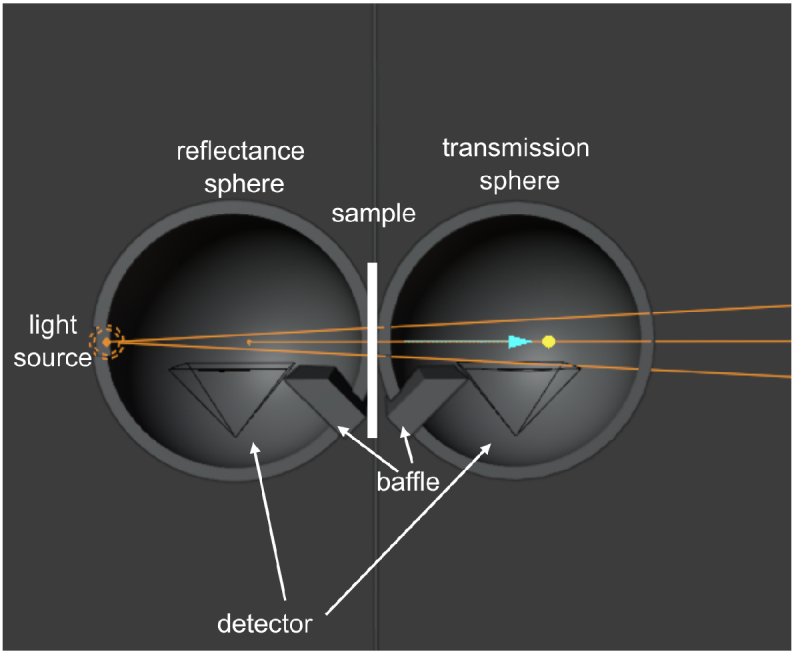
Double integrating sphere (DIS) set-up in *Blender* with light source entering a reflectance sphere, passing through a thin sample of material of interest and entering the transmission sphere. Baffles are placed to block specularly reflected sample rays.

For SFDI measurements, a reference material of known optical properties is required to correctly calibrate the system response (as discussed in Sect. 1). This requires determining the relationship between the material parameters in *Blender* and the recovered absorption and reduced scattering coefficients. This can be done directly with an SFDI system through a ‘trial and error’ approach [25] but this is imprecise and laborious. We therefore developed a more accurate approach that involves simulating a DIS system in *Blender* [36], shown in Fig. 2(a). The DIS consists of two hollow spheres, termed the ‘reflectance’ sphere and ‘transmission’ sphere, each with 100 mm diameter and 10 mm wall thickness. The material of these spheres is set to be highly reflective using the diffuse BSDF shader with 0 roughness and reflectance of 0.99 (configured by setting the colour parameter to white, with a brightness value of 0.99). The reflectance sphere has an entry port and an exit port, with the sample located at the exit port. The ports are square in shape with a 10 mm side length. The transmission sphere has only an entry port, where the sample is located, of the same shape and size as the reflectance sphere exit port. The sample has a thickness of 1 mm. The material of the sample is that of the material described in Sect. 2.1.

The input light source is a spot light of power 5 W, with a beam radius of 0.5 mm and a spot size of $6^{{}^{\circ}}$ . The light is placed at the entry port of the reflectance sphere. Cameras were placed at the base of each of the spheres to act as detectors, with all pixels summed together (i.e. integrated over the detector area) to give a power value. For our initial tests, only the red channel is considered. A baffle is placed between the sample ports and the cameras to block specularly reflected light from the sample entering the camera detector. To perform normalisation, a reflectance standard sample is simulated using the diffuse BSDF shader with roughness set to 0 and reflectance of 0.7 to improve accuracy in absorption coefficient [41]. For each captured image, the camera exposure was varied until the average intensity was approximately in the middle of the 0-255 range (i.e. 8-bit colour). This exposure was noted and corrected for using Eq. (1).

To determine the absorption and reduced scattering coefficients, a series of images is taken in the reflectance sphere and the transmission sphere, and the normalised reflectance and transmission are calculated respectively for varying sample material properties using the equations:

(2) $$M_{R}=r_{std}\frac{R_{2}(r_{s}^{\text{direct}},r_{s},t_{s}^{\text{direct}},t_{s})-R_{2}(0,0,0,0)}{R_{2}(r_{std},r_{std},0,0)-R_{2}(0,0,0,0)}$$

(3) $$M_{T}=\frac{T_{2}(r_{s}^{\text{direct}},r_{s},t_{s}^{\text{direct}},t_{s})-T_{2}(0,0,0,0)}{T_{2}(0,0,1,1)-T_{2}(0,0,0,0)}$$

where $r_{std}$ is the normalised reflectance of the reflectance standard, $R_{2}(r_{s}^{direct},r_{s},t_{s}^{direct},t_{s})$ and $T_{2}(r_{s}^{direct},r_{s},t_{s}^{direct},t_{s})$ are reflectance and transmission measurements respectively when the sample material is in place, $R_{2}(r_{std},r_{std},0,0)$ is a reflectance measurement when the standard reflectance sample previously described is in place of the material, $R_{2}(0,0,0,0)$ is a reflectance measurement when there is no sample present and the transmission sphere is removed, $T_{2}(0,0,1,1)$ is a transmission measurement when light passes straight through the reflectance sphere when no sample is present into the transmission sphere and $T_{2}(0,0,0,0)$ is a transmission measurement when the incident beam is blocked and there is no sample in the port. These normalised values were then input into an inverse adding doubling (IAD) algorithm to determine the optical properties [42].

In order to validate our SFDI recovery approaches against these DIS values, we selected 9 combinations of absorption and scattering values ranging from $\mu_{a}=0.08$ to 0.22mm^−1^ and $\mu_{s}^{\prime }=1.4$ to 6.5 mm^−1^. These values represent a very wide range of optical properties over which to evaluate our model. Using the DIS we established that these values correspond to Blender material parameters of $A_{\rho }:50-100$ and $S_{\rho }:5000-20000$ .

To evaluate the performance of SFDI, we next captured images in our SFDI set-up for these same material parameters. The system consists of a camera placed 0.5 m above the sample of interest and a 5 W spot light source, acting as the projector, placed at a $4^{{}^{\circ}}$ offset to the camera to reduce any specular reflections. The camera and projector were placed at the same height from the sample, at 0.035 m apart. The optical properties in the up-close planar geometry were calculated using two different LUTs: a Monte Carlo generated LUT and an empirically-derived LUT.

#### 2.2.2 SFDI: Monte Carlo LUT

**Fig. 3.**
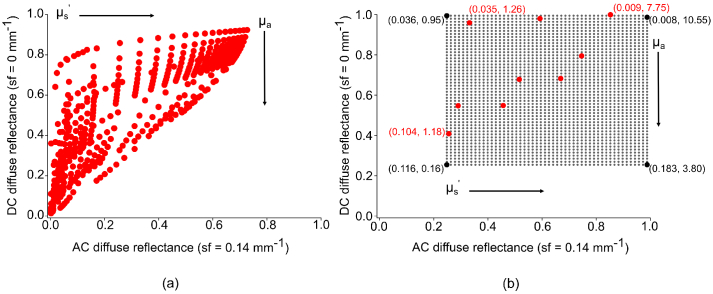
(a) DC vs AC reflectance showing values sampled for MC LUT. (b) DC vs AC reflectance showing values sampled for empirically derived LUT. Red dots represent simulated optical properties and black dots represent extrapolated sample points for larger LUT. Optical properties of selected points are displayed as $\left(\mu_{a},\mu_{s}^{\prime }\right)$ with units $mm^{-1}$ .

The Monte Carlo (MC) LUT was generated using Virtual Photonics Monte Carlo simulation software [43]. Here, we are able to sample a large range of $\mu_{a}$ and $\mu_{s}^{\prime }$ so we select a range that covers the same 9 samples tested in the IAD and also covers the range of our chosen biomedical examples of imaging Barrett’s oesophagus and squamous cell carcinoma. The ranges of the LUT are therefore $\mu_{a}=0.001$ to 0.3mm^−1^ and $\mu_{s}^{\prime }=0.1$ to 8.5mm^−1^. The spacings within this range are variable, but are depicted in Fig. 3(a). For comparison with the IAD algorithm, the optical properties of the nine material values were calculated using a reference material of $A_{\rho }=100$ and $S_{\rho }=20000$ with the corresponding reference optical properties determined from the IAD algorithm.

To simulate SCC with the optical properties obtained from literature, we configured healthy background tissue with $A_{\rho }=37$ and $S_{\rho }=2591$ , and polyps with $A_{\rho }=69$ and $S_{\rho }=2253$ . For Barrett’s oesophagus we configured the material to have $A_{\rho }=37$ and $S_{\rho }=1855$ .

Because the absorption shader used to create our material in Blender does not implement scattering, it behaves like a forward scattering material (anisotropy, g=1 ). The combination of this and the scattering shader, which does have an anisotropy setting, may create an overall ‘effective’ anisotropy, $g_{eff}$ , of the composite material. To find what $g_{eff}$ of the material is, we generated several LUTs with g values ranging from 0−0.99 , and found the material values from the IAD algorithm with g values in the same range. We then assumed $g_{eff}$ to be the point where the reduced scattering coefficient from the IAD algorithm matched the reduced scattering coefficient from the SFDI calculation.

#### 2.2.3 SFDI: Empirically-derived LUT

The empirically-derived LUT is able to correct for discrepancies between the SFDI and IAD measurements which arise from the different assumptions made in the models and can be as large as 19% [44]. To generate an empirical modulation vs reflectance LUT we used the process described by *Erickson et. al.* [32]. We started using planar samples with the same nine data points used for the IAD and captured the modulation and reflectance of these densities, and then did a first linear extrapolation using these data points to increase the LUT from 9 data points to 100 × 100 data points, improving granularity of final optical properties. This is shown in Fig. 3(b). Given the relative smoothness of the surface sampled by the original 9 points, we find this extrapolation gives reliable and consistent results for later optical property estimation including of SCC and BO samples. When applying the LUT, a further interpolation step, this time using bicubic interpolation, is carried out to determine the optical properties of a sample of interest.

### 2.3 Robust shape determination

In addition to measuring optical properties, we also reconstruct 3D shape via fringe profilometry. To do this, we consider a fringe projection pattern of the form:

(4) ψ(x,y)=sin⁡(ωy+ϕ)

where ω=2πf is the angular frequency of the projected pattern with spatial frequency f . The sinusoidal pattern must be rotated $90^{{}^{\circ}}$ from the optical property measurements so that a change in vertical height corresponds to a displacement of the projected pattern and thus a phase shift [45].

For proof-of-principle, we use a generalised approach of using 3 phase-shifted images to reconstruct height maps, though if speed is desired a single image is sufficient [46]. If the geometry of the system is precisely known, the inferred phase shift can be converted to height for each pixel in the image via the equation [33]:

(5) $$h(x,y)=\frac{l_{0}\Delta \phi (x,y)}{\Delta \phi (x,y)-2\pi f_{0}d}$$

where $l_{0}$ is the distance from the projector to the reference material, Δϕ is the phase difference between the actual phase (calculated) and the phase of the background reference plane, $f_{0}$ is the spatial frequency of the projected pattern and d is the separation distance of the projector and camera. Because of the geometrical assumptions made in mapping phase to height, this approach cannot be straightforwardly applied to non-planar geometries for shape reconstruction. In non-planar geometries, reconstruction of exact physical height could instead be approximately deduced by comparison with a reference phantom, e.g. perfectly straight tube for a lumen geometry, or by applying advanced techniques such as deep-learning [47].

### 2.4 Development of projection pattern for tubular geometry

**Fig. 4.**
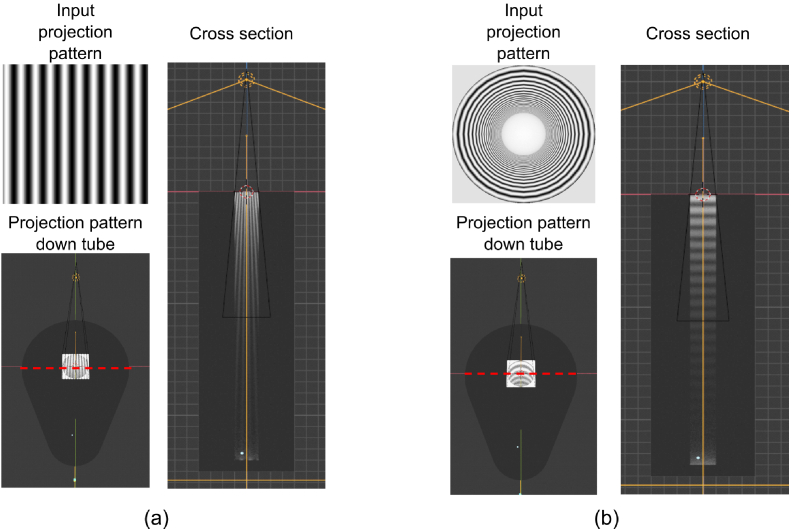
Comparing tubes with (a) planar sinusoidal pattern exhibiting varying spatial frequency along the length of the tube and (b) our novel illumination pattern with constant spatial frequency down the length of the tube. Top left insets show image being projected. Bottom left insets show view of projected pattern on top face of tube with red dashed line indicating where tube is cut to view cross section on right inset.

For *in vivo* endoscopic use, an SFDI system would need to be operated inside a tubular lumen, e.g. the gastrointestinal tract. Using our *Blender* simulation it is straightforward to explore such a situation. We began by simulating a tube of length 250 mm with an outer diameter of 80 mm and an inner diameter of 20 mm. The distal end of the tube is covered by the same material as the walls of the tube. A 120 mW spot light source was placed at a distance of 100 mm from the top of the tube and projected a 2D sinusoidal pattern down the tube. This naive approach creates a non-uniform spatial frequency pattern throughout the length of the tube which makes reconstructing accurate optical properties challenging (see Fig. 4(a)). Therefore, we developed a process to create a more suitable illumination pattern for other imaging geometries and demonstrated for the test case of a tube. First, the material of the tube was set to be highly reflective using a pre-existing material node of diffuse BSDF with a roughness of 0 and a shade of pure white. Next, the surface of the tube was ‘unwrapped’ within *Blender* using the UV mapping tool, resulting in a flattened map of the inside of the tube. A sinusoidal pattern of the desired phase and spatial frequency was then applied to this flat surface. Once applied, the material is then wrapped, such that the inside of the tube now has a uniform spatial frequency throughout its length. 1 W light sources were placed equally throughout the tube such that the illumination intensity is uniform looking down the tube at the top. Here, we evenly distributed 40 point sources down the 250 mm tube. A camera placed 110 mm above the top of the tube then captured an image of the concentric circle illumination pattern. This image was then exported to *Python* where a normalisation was applied to ensure that the sinusoid pixel values vary across the maximum range for projection ( 0−255 ). This process was carried out for sinusoidal patterns of a fixed spatial frequency at 3 different phase shifts.

The normalised images of the patterned tube are used as the new projection patterns, which are projected onto the tube with a 5 W light source, shown in Fig. 4(b). This process can be considered a ‘pre-distortion’ of the projected pattern to produce more uniform spatial frequencies and could alternatively be computed using analytically-derived formulae, or by direct inverse computation using a ray-tracing engine. These modified projection patterns can then be used for SFDI imaging as there is a now a uniform spatial frequency pattern within the geometry length.

However, the tubular geometry inherently allows less light to reach the distal end of the tube and less light to be reflected back as only a small range of angles can escape the tube via the opening. The projector placement, at a large angle to the normal of the tube surface, also creates different incidence angles along the length of the tube. It is therefore necessary to apply the empirically derived LUT approach in this case to account for these effect. Further, to account for variation along the tube, we developed a longitudinal sectioning approach: the tube is divided in 5 different longitudinal subsections, each with its own LUT. The five sections were selected as regions that showed a mean intensity difference >10 relative to other sections.

## 3. Results

### 3.1 Material simulation

By repeated DIS simulation, we found that appropriate parameter ranges to produce the desired optical properties were $50\leq A_{\rho }\leq 100$ and $5000\leq S_{\rho }\leq 20000$ . We selected material of $A_{\rho }=100$ and $S_{\rho }=20000$ with optical properties $\mu_{a}=0.217{\text{ mm}}^{-1}$ and $\mu_{s}^{\prime }=5.94{\text{ mm}}^{-1}$ to be the reference material for the SFDI measurements.

**Fig. 5.**
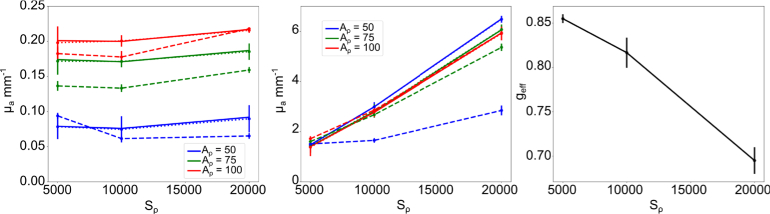
(a) Absorption and (b) reduced scattering coefficient vs scattering density, $S_{\rho }$ , calculated for varying absorption densities, $A_{\rho }$ , via IAD algorithm (solid line), SFDI Monte Carlo LUT (dashed line) and SFDI empirically derived LUT (dotted line). The error bars represent the standard deviation across the calculated 500×500 pixel optical property map. (c) Effective anisotropy found to correct for reduced scattering coefficient. Error bars represent standard deviation across $g_{eff}$ over all bulk material absorption densities

The results from the SFDI measurements are compared with the DIS results in Fig. 5(a and b). We note that there are discrepancies between the absorption and reduced scattering results from the IAD and the SFDI Monte Carlo LUT calculations, with an average standard error of 16% and 18% respectively. This is caused in part because the different methodologies rely on different assumptions and have different sources of error. Experimental studies typically find up to 19% observed discrepancy in optical properties [44]. To examine the impact of inaccuracies in anisotropy caused by the mixing of Blender shaders, we plot $g_{eff}$ in Fig. 5(c). We observe that for low scattering values, where absorption shader is dominant, $g_{eff}$ is greater than the anisotropy value of 0.8 specified in the scattering shader settings, but decreases to 0.7 for high scattering. This characterisation could be expanded to compute $g_{eff}$ for a wider range of scattering values and hence increase accuracy of simulation. To account for these discrepancies, we introduce the empirically-derived LUT with resultant calculated optical properties displayed in Fig. 5(a) and (b).

### 3.2 Simulation of typical gastrointestinal conditions in up-close planar geometry

**Fig. 6.**
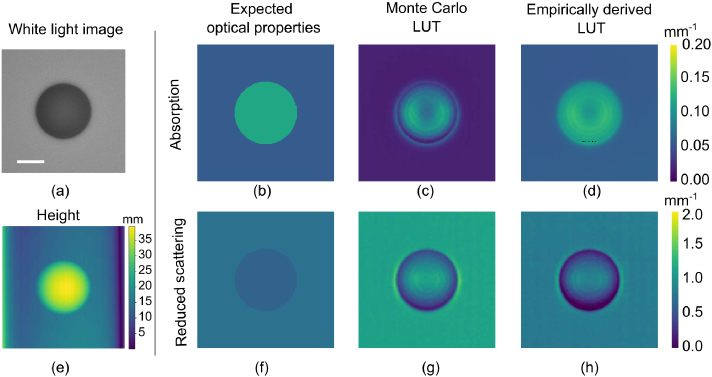
Simulated squamous cell carcinoma (SCC) as a spheroid on a background of healthy oesophageal tissue (HT) showing (a) white light image and (e) reconstructed height map with (b) expected absorption coefficient where, $\mu_{a,SCC}/\mu_{a,HT}\approx 2$ , (c) $\mu_{a}$ recovered with MC LUT (d) $\mu_{a}$ recovered with empirically derived LUT (f) expected reduced scattering coefficient, where $\mu_{s,SCC}^{\prime }/\mu_{s,HT}^{\prime }\approx 0.85$ , (g) $\mu_{s}^{\prime }$ recovered with MC LUT and (h) $\mu_{s}^{\prime }$ recovered with empirically derived LUT. Scale bar = 20mm.

Figure 6 shows the optical property and height maps generated for a 80 mm diameter simulated polyp, with an absorption coefficient higher than that of surrounding healthy tissue and a reduced scattering coefficient lower than that of surrounding healthy tissue, simulating squamous cell carcinoma. Figure 6(e) shows a successful height map generation from fringe profilometry measurements. Figure 6(c) and (g) demonstrate successful recovery of optical properties using the Monte Carlo LUT. Figure 6(d) and (h) demonstrate successful optical property recovery using the empirically derived LUT. The empirically derived LUT produces results closer to the expected values, which is because it accounts for discrepancies in our tissue simulation as described earlier. However,the Monte Carlo LUT still provides high contrast between the squamous cell carcinoma and background, which is arguably more important for wide-field diagnostic applications. We note that because the surface profile information is available, the optical property accuracy may be improved by the addition of surface profile correction for optical property determination [45].

**Fig. 7.**
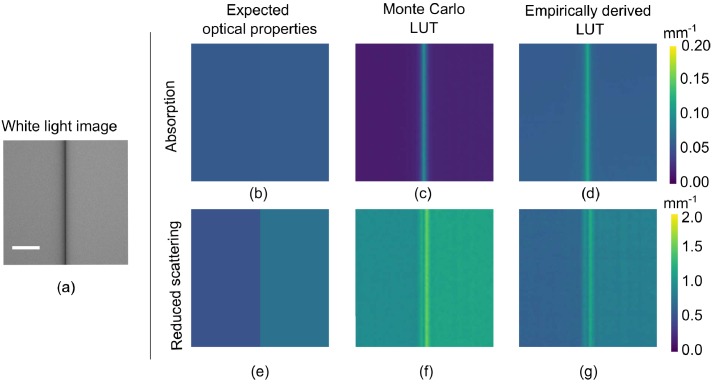
Simulated Barrett’s Oesophagus (BO) with mild chronic inflammation (left half of sample) adjacent to healthy oesophageal tissue (right half of sample) showing (a) white light image (b) expected absorption coefficient, where $\mu_{a,BO}/\mu_{a,HT}\approx 0.99$ , (c) $\mu_{a}$ recovered with MC LUT and (d) $\mu_{a}$ recovered with empirically derived LUT (e) expected reduced scattering coefficient, where $\mu_{s,BO}^{\prime }/\mu_{s,HT}^{\prime }=0.68$ , (f) $\mu_{s}^{\prime }$ recovered with MC LUT and (g) $\mu_{s}^{\prime }$ recovered with empirically derived LUT. Scale bar = 20mm.

Figure 7 shows the optical property maps generated for a segment of Barrett’s oesophagus next to a segment of healthy oesophageal tissue. The tissue properties are designed to exhibit similar absorption coefficients, while the reduced scattering coefficient of the simulated BO is less than that of the adjacent healthy oesophageal tissue. Figure 7(c),d,f and g show these optical properties are recovered as expected, demonstrating the capability of the simulation system to differentiate between tissue types. We note that at the intersection region of the two simulated tissue types, there is a spike in both the optical properties, which results from effects at the interface and a small air gap that is present.

### 3.3 Effect of camera angle

We simulated and imaged a planar sample of healthy oesophageal tissue with the projector at a $4^{{}^{\circ}}$ and $20^{{}^{\circ}}$ angle to the camera. We noted differences of just 12% and 4% in the AC and DC modulation amplitudes respectively, corresponding to relative error in calculated absorption and reduced scattering properties of 3% and 11% respectively. We chose the smaller angle so as to be compatible with realistic miniaturisation of SFDI systems operating in space-constrained environments. Previous experimental work has shown SFDI systems can work with small camera projector angles of $8^{{}^{\circ}}$ [48].

### 3.4 Simulation of optical property variation in tubular geometry

**Fig. 8.**
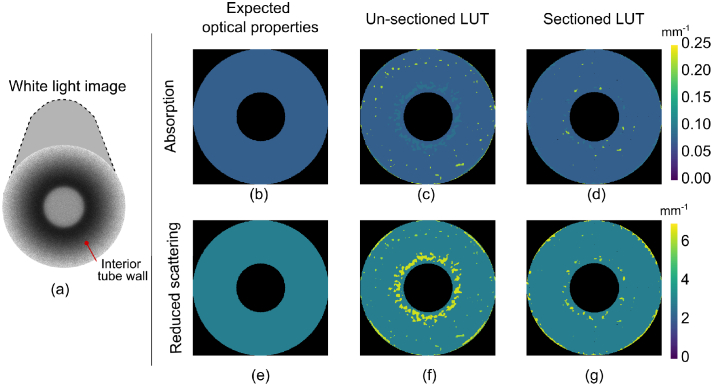
Comparison of sectioned and un-sectioned empirically derived LUT for tube wall material of $\mu_{a}=0.076\;{\text{mm}}^{-1}$ and $\mu_{s}^{\prime }=2.99\;{\text{mm}}^{-1}$ (a) white light image of tube (b) expected absorption coefficient, $\mu_{a}$ , (c) simulated $\mu_{a}$ using un-sectioned LUT (d) simulated $\mu_{a}$ using *sectioned* LUT (e) expected reduced scattering coefficient, $\mu_{s}^{\prime }$ , (f) $\mu_{s}^{\prime }$ simulated using un-sectioned LUT, (g) $\mu_{s}^{\prime }$ simulated from *sectioned* LUT. Tube inner diameter = 20mm.

**Fig. 9.**
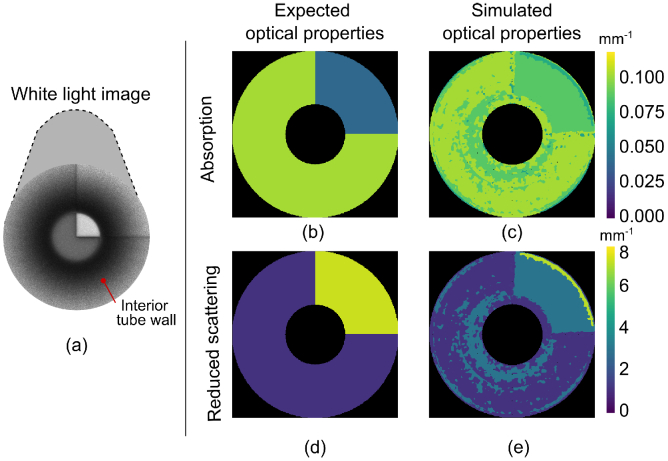
Imaging different material types within complex geometry, analogous to a lumen showing (a) white light image of the tube (b) expected absorption coefficient of tube material (c) simulated absorption coefficient (d) expected reduced scattering coefficient of tube material and (e) simulated reduced scattering coefficient. Tube inner diameter = 20mm.

We next used our custom projection pattern modified for a tube to produce Fig. 8 and Fig. 9. The optical property maps have a quantized appearance due to the use of nearest-neighbour interpolation, which we find increases robustness for points outside the convex hull of the LUT. A larger LUT could be generated with more sample images from Blender to mitigate this effect using bicubic interpolation. We observe that the simulated AC modulation amplitude was higher than expected, which may be due to the high incidence angle of the light creating substantially different scattering and reflectance behaviour. Though we correct for this to a large degree using empirically-derived LUTs, there is still a residual increase in AC modulation amplitude and an offset in reduced scattering coefficient. Significant improvement was achieved when longitudinally sectioning the LUT shown in Fig. 8(d) and 8(g). We calculated, over six varying material values, that the sectioned LUT method reduced the calculated absorption coefficient relative error from 5% to 2% and reduced the calculated reduced scattering coefficient relative error from 5% to 2% compared to the SFDI global LUT.

Finally, to simulate detection of disease inside a lumen we simulated a tube with one quadrant exhibiting a large variation in optical properties compared to the remaining three quadrants. The results are shown in Fig. 9 and we observe a distinct difference in material properties in the top right quadrant, as expected.

## 4. Discussion

These results demonstrate the capability of our *Blender* SFDI simulation system to recreate various tissue types in various shapes and imaging geometries, and then reconstruct these optical properties using standard SFDI algorithms. Existing software such as *OptogenSIM*[49], *FullMonte*[50] and *ValoMC*[51] offer Monte Carlo simulations in biologically relevant samples, but suffer from a variety of limitations including incapability to generate realistic, complex sample geometries within the software and lack of simulation of lighting conditions or camera positions. The presented SFDI simulation model can overcome many of the limitations of existing software by enabling custom configuration of illumination source and camera position and orientation, spatial frequency, and illumination pattern. Our model could also allow exploration of some typical sources of error in SFDI. SFDI can have various sources of errors arising from assumptions made with selected light propagation model, differences in optical properties dependent on depth, divergence of the projection beam, how the spatial frequency may change with distance from projector to sample, and different probing depths achieved by different spatial frequencies [52,53].

The introduction of these real-world artefacts will help to test the limitations and robustness of new SFDI system design. We therefore envisage our Blender system could accelerate development of novel SFDI systems for applications such as endoscopy or LIDAR, by speeding up initial development and testing of new imaging configurations, lighting conditions and illumination patterns. Another potential application of this system could be to generate large SFDI data sets that may be used in lieu of or in addition to experimental data. Such data sets could be used to improve optical property uncertainty measurements by creating large look up tables for specific system setups [54] or to train deep-learning SFDI recovery systems [26,55].

There are a few key limitations of our model. The first is the discrepancy observed between DIS and SFDI results, particularly at low absorption values. We note that previous work has shown a difference in DIS and SFDI absorption coefficients of 19% [44], even when using a more accurate method of determining the absorption coefficient than the conventional IAD algorithm used for the DIS. It is also well known that DIS measurements can have poor accuracy for absorption recovery [41]. The error we observe is consistent with this so may be a result of the different underlying assumptions of the two approaches. The cross-coupling between absorption and scattering may arise in part because increased absorption reduces the accuracy of scattering measurements as there will be fewer ‘scattering’ events simulated for each ray before it is absorbed. The effect observed here is comparatively small and so for the purposes of designing SFDI systems may be neglected.

The second limitation is variation in effective anisotropy as a function of scattering due to the way shaders are combined. However, we have shown that this can be characterised and so look up tables could have an extra dimension added to them containing effective anisotropy, enabling this parameter to be controlled independently.

The third limitation is the presence of some artefacts in the tubular geometry configuration. We speculate these may be caused by light reflecting off multiple surfaces before reaching the camera, or are residual errors due to large, spatially varying incidence angles that are not entirely corrected by our empirical LUT approach. Further work is required to increase accuracy, perhaps by the addition of more longitudinal sections in the empirically derived LUT. Since the position of our camera and projector are not fixed, they could also be advanced into the tube to characterise how optical property accuracy changes when features such as polyps move closer. The animation feature of blender could be used to straightforwardly simulate this scenario, producing multiple video frames as the camera and projector move along the tube.

The final limitation is operation in only 3 wavelengths: red, green and blue. This is a fundamental limitation of Blender, but scattering at other wavelengths could be simulated by adjusting the material scale to change the scattering length scales. However, our tool is intended as a geometrical design tool for SFDI systems that should be used in combination with Monte Carlo simulators for more accurate design at other wavelengths.

## 5. Conclusion

We have shown the capability of the open-source graphics software *Blender* to be used to simulate SFDI and fringe profilometry systems. The software enables the simulation of typical gastrointestinal conditions with specific absorption and reduced scattering coefficients in tubular imaging geometries relevant for endoscopy in the gastrointestinal tract. We have shown simulation of objects of specific shape, size and optical properties and successful imaging of these objects to recover maps of absorption, scattering and height. We anticipate our results will aid in the design of future SFDI systems, e.g. miniaturised systems, by enabling the testing of different illumination geometries and patterns.

## Data Availability

Data underlying the results presented in this paper are available in Ref. [56]
